# Mouse intestinal microbiome modulation by oral administration of a GABA-producing *Bifidobacterium adolescentis* strain

**DOI:** 10.1128/spectrum.02580-23

**Published:** 2023-11-22

**Authors:** Héctor Tamés, Carlos Sabater, Félix Royo, Abelardo Margolles, Juan Manuel Falcón, Patricia Ruas-Madiedo, Lorena Ruiz

**Affiliations:** 1 Department of Microbiology and Biochemistry of Dairy Products, Instituto de Productos Lácteos de Asturias-Consejo Superior de Investigaciones Científicas (IPLA-CSIC), Paseo Río Linares s/n, Villaviciosa, Asturias, Spain; 2 Functionality and Ecology of Beneficial Microbes (MicroHealth) Group, Instituto de Investigación Sanitaria del Principado de Asturias (ISPA), Oviedo, Asturias, Spain; 3 Exosomes Laboratory, Center for Cooperative Research in Biosciences (CIC bioGUNE), Basque Research and Technology Alliance (BRTA), Derio, Spain; 4 Centro de Investigación Biomédica en Red de Enfermedades Hepáticas Y Digestivas (CIBERehd), Madrid, Spain; 5 IKERBASQUE, Basque Foundation for Science, Bilbao, Spain; University of Washington, Seattle, Washington, USA

**Keywords:** *B. adolescentis*, GABA, microbiome modulation

## Abstract

Emerging evidence suggests that gut microbes can significantly contribute to central nervous system (CNS) well functioning through several gut microbiome-brain signaling mechanisms, among which the production of neurotransmitters by commensal microbes is very relevant. Hence, there is increasing interest in developing probiotics with capacity to deliver, locally within the gut ecosystem, neurotransmitters. The gamma-aminobutyric acid (GABA) is the main inhibitory neurotransmitter in the CNS, and its disbalance has been associated with numerous disorders, including depression or anxiety. Furthermore, some commensal gut microorganisms are capable to produce GABA from its precursor, glutamate, whose concentration is generally higher in patients from disorders such as fibromyalgia, chronic fatigue, and pain. Therefore, we postulate that the administration of GABA-producing probiotic microorganisms may contribute to ameliorate conditions related to high glutamate/low GABA concentrations. In a prior work, we demonstrated a significant reduction of serum glutamate concentration in mice following 2-week administration of a GABA-producing *Bifidobacterium adolescentis* strain, IPLA60004. Herein, we further investigate the impact that the probiotic administration may have on the gut microbiome composition and metabolism. Remarkably, the gut microbiota modulation observed was different in animals receiving the GABA-producing strain, IPLA60004, as compared to animals receiving a closely related strain without GABA-producing ability. Genera of commensal and beneficial microorganisms, including *Lactobacillus, Roseburia,* and novel *Lachnospiraceae* genera, reached significantly higher representation at late intervention points in the fecal microbiota of animals receiving the GABA-producing strain, suggesting that some of the physiological effects of the probiotic administration may be linked to specific gut modulation effects.

**IMPORTANCE:**

The gut microbiome-brain communication signaling has emerged in recent years as a novel target for intervention with the potential to ameliorate some conditions associated with the central nervous system. Hence, probiotics with capacity to produce neurotransmitters, for instance, have come up as appealing alternatives to treat disorders associated with disbalanced neurotransmitters. Herein, we further deep into the effects of administering a gamma-aminobutyric acid (GABA)-producing *Bifidobacterium* strain, previously demonstrated to contribute to reduce serum glutamate levels, in the gut microbiome composition and metabolic activity in a mouse model. Our results demonstrate that the GABA-producing strain administration results in a specific pattern of gut microbiota modulation, different from the one observed in animals receiving non-GABA-producing strains. This opens new avenues to delineate the specific mechanisms by which IPLA60004 administration contributes to reducing serum glutamate levels and to ascertain whether this effect could exert health benefits in patients of diseases associated with high-glutamate serum concentrations.

## INTRODUCTION

The gamma-aminobutyric acid (GABA) is the main inhibitory neurotransmitter in the central nervous system (CNS) of mammals and is synthesized *in vivo* from glutamic acid. Insufficient GABA has been related to different nervous system pathologies such as anxiety, depression (1, 2), schizophrenia (3), or fibromyalgia (4), with sex-related differences associated with both GABA metabolism and disease risk (4 – 6). Accordingly, GABA supplementation has demonstrated benefits for treating anxiety, depression, and preventing neuronal death or damage after ischemia reperfusion in several *in vitro, in vivo,* and clinical trials (7 – 10). Not only the effects of GABA have been circumscribed to the CNS, like anti-hypertensive, anti-diabetic, anti-inflammatory, or anti-oxidant effects (11), but also at intestinal level, it demonstrated to reduce visceral sensitivity (12), ameliorate local inflammation (13), modulate the motility (14), or improve the course of some severe pathogenic infections (15). Accordingly, oral administration of GABA has been deemed capable of exerting effects locally within the gut but also at the systemic level through exosome production (1, 16).

In recent years, it has been demonstrated that several gut microbiota members can significantly contribute to the production of several neuroactive compounds at the intestinal level (17). Among these, GABA production has been described in several *Bacteroides, Ruminococcus, Bifidobacterium, Enterococcus,* or lactobacilli species, among others (18 – 20). In this line, it is worth highlighting that some disorders associated with deficient GABA levels have also been associated with gut microbiome alterations. As some examples, depression and autism spectrum disorders have been linked with gut microbiome underrepresentation or even depletion of microbial taxa, including reduced representation of GABA-producing pathways (21, 22). In a cohort of fibromyalgia patients, gut microbiome and metabolome analyses also demonstrated a lower abundance of *Bifidobacterium* and lactobacilli species associated with elevated levels of GABA precursors, glutamate, and glutamine, in serum (4).

Production of neurotransmitters, such as GABA, by the gut microbiota may contribute to host GABA levels and can significantly impact host physiology at local and systemic levels (23). This has opened an area of research into the gut microbiota as a target for intervention in disorders associated with deficient neurotransmitter levels. Hence, the administration of GABA-enriched foods and/or (probiotic) bacterial cultures capable to deliver GABA into food matrixes or locally, within the gastrointestinal tract (24), has been pointed as an appealing alternative to ameliorate conditions associated with GABA deficiencies and microbiome disbalances. In this regard, lactobacillus has been one of the bacterial groups most investigated as GABA-producing cultures for probiotic and food applications (25 – 28).

In a previous work, we have demonstrated GABA production in a collection of *Bifidobacterium adolescentis* strains *in vitro* (19). Remarkably, *B. adolescentis* species holds the qualified presumption of safety (QPS) status by the European Food Safety Authority (EFSA), including several strains with recognized probiotic traits, and the genus frequently appears underrepresented in the gut microbiota in several disorders associated with GABA deficiencies such as fibromyalgia (4). Related to these previous observations, this work exposes further data from a preclinical investigation using a rodent animal model (mouse) of delivering a viable GABA-producing *Bifidobacterium adolescentis* strain. In a prior work, the influence of the administration on target metabolites at brain and serum level was reported. Herein, we deep into the influence of the probiotic administration on the gut microbiome which was explored through target analysis of relevant metabolites in feces, together with metataxonomic analysis of the intestinal microbiota through 16S rRNA sequencing of fecal and colonic content samples.

## MATERIALS AND METHODS

### Bacterial strains and growth conditions

Two *B. adolescentis* strains were used in this work. The strain *B. adolescentis* IPLA60004, from here onward IPLA60004, belongs to IPLA-CSIC culture collection. IPLA60004 was previously isolated from a human colonic biopsy and was demonstrated to have a high capability to convert the GABA precursor monosodium glutamate (MSG) into GABA (19, 29). The strain *B. adolescentis* LMG10502^T^, from here onward LMG10502^T^, was purchased from the Belgium culture collection (BCCM/LMG Bacteria Collection, Belgium) and was previously demonstrated to lack *gad* genes, and hence, the capacity to produce GABA. The strains were routinely grown in MRS_c_ medium [MRS (Biokar, France) supplemented with 0.25% (wt/vol) L-cysteine hydrochloride monohydrate (Sigma-Merck, Germany)]. Stocks stored at −80°C were resuscitated in agar-MRS_c_ in anaerobic jar with anaerocult A (Merck, Germany) at 37°C for 2 days. Isolated colonies were inoculated in 50 mL of MRS_c_ broth and incubated under the same conditions for 20 ± 1 h to obtain the bifidobacterial cultures.

### *In vivo* study design and sample collection

All animal experimentation was conducted in accordance with Spanish guidelines for the care and use of laboratory animals, and protocols were approved by the CIC bioGUNE Institute and the regional Basque Country ethical committee (ref. P-CBG-CBBA-1521) as previously described, using sex-segregated groups (30). Briefly, C3H/HeJ mice were provided by the Jackson Laboratory (Bar Harbor, Ma, USA) at the age of 9 weeks. After 1-week adaptation at the animal facility, in an environmentally controlled room and provided with standard diet and water *ad libitum*, the 24 animals were distributed in 6 cages of 4 animals, forming 3 groups of males and 3 groups of females for each treatment. All the animals were fed daily by oral gavage, during 14 days, with sterilized milk (vehicle group), *B. adolescentis* IPLA60004 strain (probiotic group), or the reference *B. adolescentis* LMG10502^T^ strain (control group). At days 0, 2, 4, 5, 7, 9, 11, 12, and 14, animals were weighed, and fecal samples were obtained to monitor viable counts of *Bifidobacterium* spp. by serial dilution and colony plating in TOS-propionate agar and lithium mupirocin (TOS-MUP) (Merck). The remaining fecal pellets collected at each sampling day were frozen at −80°C until further analysis. Finally, blood samples were taken every 7 days. At the end of the experimental period, animals were sacrificed, and blood samples were taken and deep frozen immediately for metabolomic analysis, and cecum contents were collected for microbiome analyses.

### Preparation of *Bifidobacterium adolescentis* suspensions for oral administration

Both strains used in this work (control, LMG10502^T^, and IPLA60004) were grown daily throughout the experimental intervention period, on de Man-Rogosa-Sharpe (MRS, Biokar, France) broth supplemented with 0.25% L-cysteine (Merck), MRS_c_ hereafter. Bacterial cultures that were fed to the animals were prepared daily along the experimental intervention period as follows. Briefly, overnight grown cultures were washed once with sterile PBS solution and concentrated 10 times in sterilized milk, which was prepared by dissolving skimmed milk powder (BD-Difco, USA) (11% wt/vol) in water and autoclaving (121°C, 15 min). These suspensions were used for bifidobacterial enumeration by plating serial dilutions (made in Ringer ¼, Merck) on agar-MRS_c_. After incubation in the standard conditions, the logarithmic colony-forming units (CFU/mL) of the bifidobacterial suspensions were 9.20 ± 0.36 and 9.23 ± 0.32 for strains LMG10502^T^ and IPLA60004, respectively. Every day, 100 µL of fresh bacterial suspensions in milk, as well as the placebo (sterilized milk), was administered to the animals through oral cannula, thus groups administered with probiotic strains received a dose of about 10^8^ CFU per day.

### Glutamate/GABA determination in feces and colonic contents

Glutamate and GABA concentrations in feces and colonic contents were determined by high performance liquid chromatography (HPLC) coupled with a photodiode-array (PDA) detector (HPLC-PDA) on fecal waters following diethyl ethoxymethylenemalonate (DEEMM, Sigma-Aldrich) derivatization according to previously described procedures (19). First, samples were weighed and diluted with 20 volumes of PBS, using Stomacher for homogenization. Then, free amines and amino acids were first extracted using acidification with hydrochloric acid and filtrated using 3-kDa cutoff Amicon filtering units (Millipore-Merck). Finally, extracted samples were subjected to DEEMM derivatization and analyzed by HPLC-PDA as previously described.

Differences between male and female animals were first assessed statistically using two-way ANOVA, and when no differences were detected, treatment groups including both genders were compared using one-way ANOVA.

### DNA extraction and 16S rDNA sequencing

In order to extract total DNA from fecal samples and colonic contents prior to 16S rDNA sequencing, previously published (31) “Qia-Ez DNA extraction” protocol was used with minor modifications. Briefly, the protocol consisted of three sequential steps, including mechanical and enzymatical cell disruption, followed by DNA clean-up, and purification. For the mechanical cell disruption, samples were weighed and mixed with 300 µL of sterile PBS for homogenization in Stomacher, using a 2-min step. Homogenates were then transferred to vials containing 0.4 g of 0.1 mm zirconium-silica beads and one glass pearl. Cells were disrupted in a Fast-Prep-24 instrument (MP Biomedicals, Fisher Scientific, USA) by the application of three pulses of 1 min at maximum speed followed by 1 min of rest in ice. Subsequently, enzymatic lysis was performed by the addition of a lysis buffer including 600 kU/mL lysozyme, 100 U/mL mutanolysin, and 3 U/mL of lysostaphin, and samples were incubated at 37°C for 1 h with agitation every 20 min. Finally, DNA was cleaned up and purified by employing the QIAGEN Fast DNA Stool Kit (QIAGEN, Germany) following manufacturer’s instructions.

Isolated DNA was quantified using DNA high-sensitivity assay in a Qubit fluorimeter (both from Thermo Fisher, USA). 16S rRNA sequencing was performed by amplifying V3 regions using the primer’s pair Probio_Uni and /Probio_Rev, as previously described (29), and samples were submitted to 2  ×  250 bp paired-end sequencing in an Illumina MiSeq System instrument (Illumina) at GenProbio S.R.L. (Italy). Sequence reads were quality filtered by the Illumina software and then trimmed, and filtered sequences were processed with a custom script based on the QIIME2 (v.2021.8) software suite (32 – 34). Briefly, quality control filtering was performed, keeping sequences with a mean sequence quality score >20 and a length between 140 and 400 bp, and retained reads were classified to the lowest possible taxonomic rank using the reference database SILVA 138 release. Microbiome-specific data analysis methods at phylum, family, and genus level were computed on R (v.4.2.2.) as follows. Alpha diversity metrics of fecal and colonic content samples were calculated using Microbiome R package (35), and beta-diversity analysis was performed using the Bray-Curtis dissimilarity method implemented in Phyloseq R package (36). Principal coordinate analysis (PCoA) of microbiome composition was computed using Microbiome R package (35).

Then, multiple statistical methods designed for microbiome analysis (aldex, ANCOM. ANCOMBC, LEfSe, and metagenomeSeq) implemented in microbiomeMarker R package (37 – 42) were performed. Taxonomic clades classified as significantly differential microbes (microbiome markers) by any of these methods were selected for further analysis. In addition, clades showing abundance differences higher than 5% in all samples from different groups were also considered. In this regard, changes in fecal microbiota samples from different groups (vehicle group, control strain, and probiotic strains) were determined at different times (0, 2, 4, 5, 7, 9, 11, 12, and 14 days). Similarly, differences among colonic content samples (time 14 days) from different groups (vehicle, control strain, and probiotic strains) were also determined.

Finally, statistical correlations between taxonomic clades showing differences among groups and biochemical parameters [short-chain fatty acids (SCFAs), GABA, and glutamine levels] were calculated and expressed as Pearson correlation coefficients using base R (v.4.2.2.) functions.

### Short-chain fatty acid determination in feces and colonic contents

SCFAs (acetic, propionic, butyric, isobutyric, valeric, isovaleric, and caproic acids) were determined by gas chromatography (GC) with in flame ionization detector (FID) (GC-FID) on fecal waters as previously described (34). Briefly samples were diluted and homogenized in PBS, and fecal waters were mixed with 4.5 volumes of methanol, 0.5 volumes of 20% formic acid, and 0.5 volumes of 2-ethylbutyric acid (Sigma, Aldrich), used as an internal standard. After centrifugation, SCFAs were analyzed in supernatants using a system composed of a 6890 GC injection module (Agilent Technologies, USA) with an HP-FFAP (30 × 250 × 0.25) column (Agilent Technologies) and a flame ionization detector (FID). Data acquisition and processing were performed using ChemStation Agilent software (Agilent Technologies). Samples were analyzed in duplicate.

### Statistical analysis

Data treatment, figures, and statistical analysis were carried out in Prism 7.0 (GraphPad, USA) and R (v.4.2.2.).

## RESULTS

### Bifidobacterial recovery from fecal and colonic content samples

**Fig 1:**
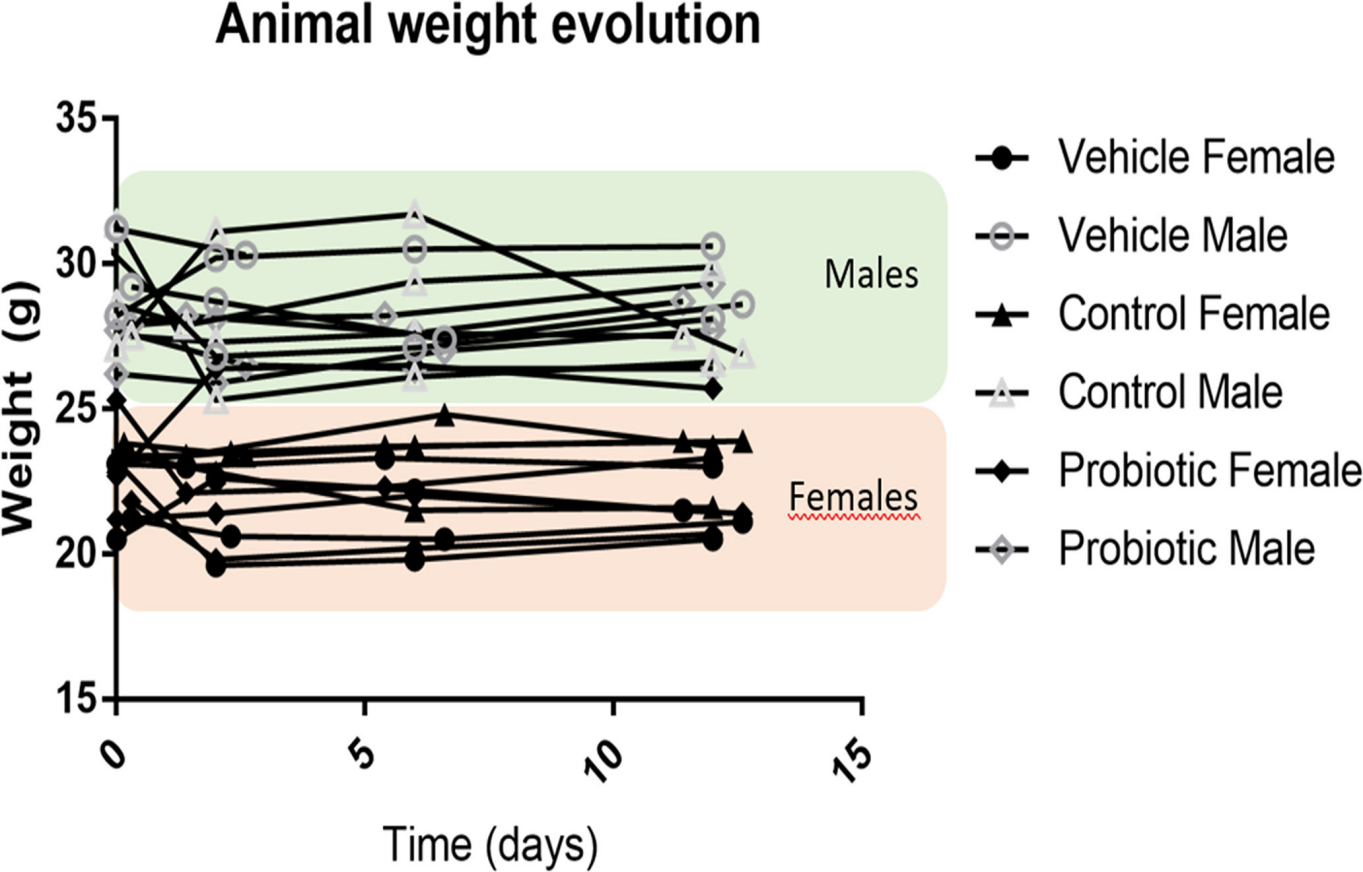
Animal weight evolution over time. Males are represented in gray unfilled geometrical forms according to treatments, whereas females are represented as black filled forms. Gender influence is highlighted by green area for males and orange area for females, where most of the measurements can be found. A total of 24 animals are represented (4 mice per treatment and gender).

Bifidobacterial culture administration did not significantly impact growth parameter in the animal groups during the experimental period (Fig. 1). Besides, fecal samples plating at the beginning of the experiment demonstrated the absence of recoverable bifidobacterial cells, whereas along the intervention period (days 2–14), an average of 5.21 ± 0.38 and 5.15 ± 0.76 logarithmic CFU/mL of bifidobacterial cells was recovered in the control groups (male and female groups, respectively), and an average of 5.46 ± 0.65 and 5.29 ± 0.54 logarithmic CFU/mL of bifidobacterial cells was recovered in the probiotic groups (male and female groups, respectively) (30). No significant differences were detected in the level of recoverable viable bifidobacterial cells for the two bifidobacterial strains administered, LMG10502^T^ (control) and IPLA60004 (probiotic), neither between male and female groups.

In order to further investigate the possible modulation that the strains administration may have on the intestinal microbiome, a metataxonomic profiling of the gut microbiota was performed (longitudinally along the intervention, from fecal samples; and at the end of the experimental period, from individual colonic contents). Besides, target metabolites including short-chain fatty acids, GABA, and glutamate were determined in these samples and correlated to the data obtained from serum and brain tissues and included in a prior publication (30). When no differences were observed among male and female animals, data are presented jointly without animal’s segregation by sex.

### Metabolite determination in feces and colonic content samples

**Fig 2:**
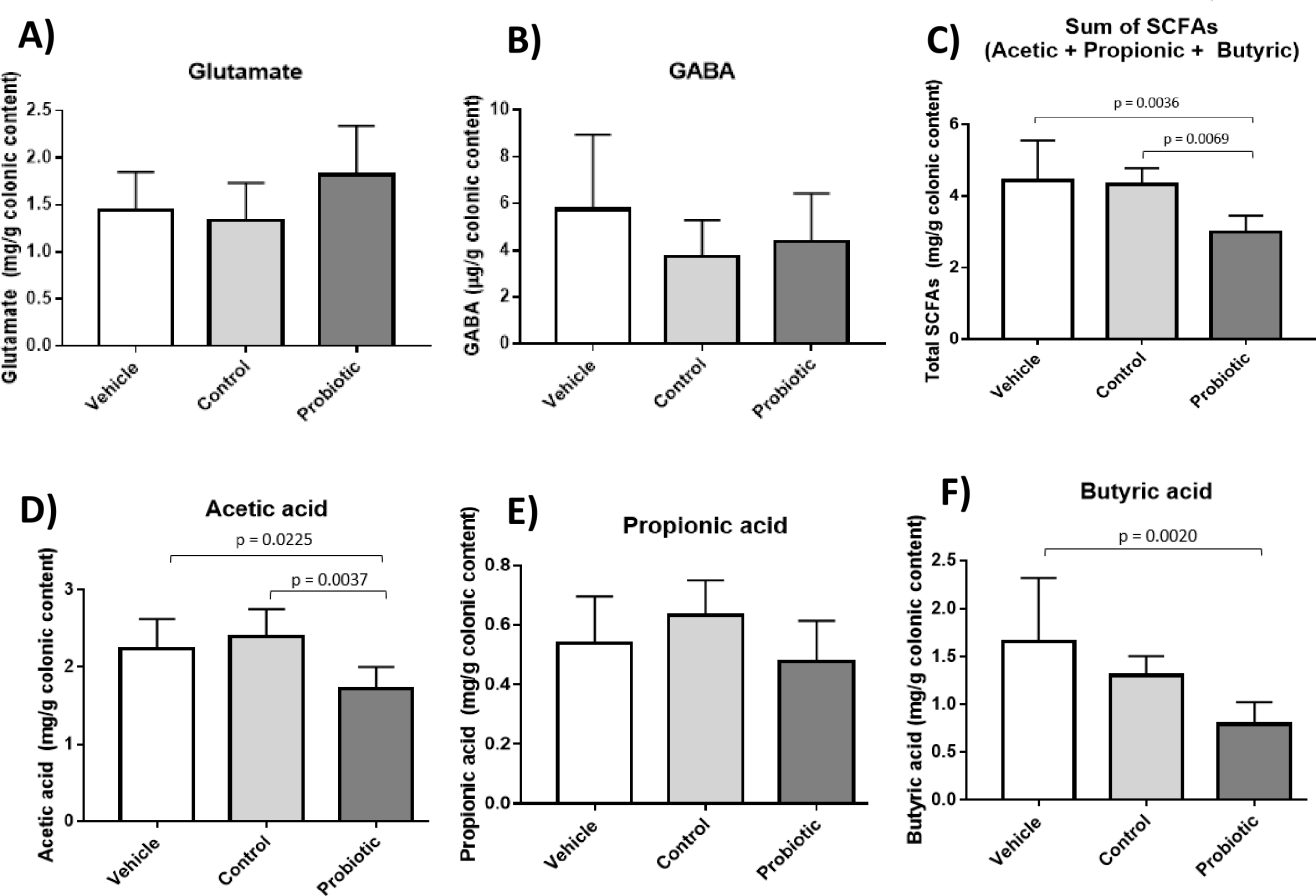
Metabolites measured in feces of animals representing glutamic acid (**A**); GABA (**B**); sum of acetic, propionic, and butyric (**C**); or individual acetic (**D**), propionic (**E**), and butyric (**F**) acids. Group comparison was carried out using one-way ANOVA. When applicable, statistically different groups were compared using Tukey’s multiple comparisons test. Brackets indicate *P*-values <0.05 for groups compared. Means represent at least seven independent animals.

In the colonic contents, determination of the concentration of GABA and its precursor, MSG, did not exhibit any significant differences among treatment groups, neither between male and female animals within treatment groups. MSG concentrations determined in colonic contents were in the range of 1.33–1.82 mg/g of colon content, whereas GABA concentrations determined were in the range of 3.7–5.7 µg/g of colon content. In relation to the determination of the major SCFA, the probiotic group exhibited a lower concentration of total SCFA, attributed to a reduced representation of acetic and butyric acids in colonic contents (Fig. 2). No significant differences were observed in the level of propionic acid among groups. In fecal samples, no significant differences were observed for any of the metabolites determined among groups, neither among male and female groups (data not shown).

### Longitudinal changes of the fecal microbiota composition through 16S rRNA profiling

With the aim of gaining a better understanding of the possible gut microbiota modulating properties of the administration of the GABA-producing *B. adolescentis* strain, a metataxonomic analysis of fecal and colonic samples was carried out.

**Fig 3:**
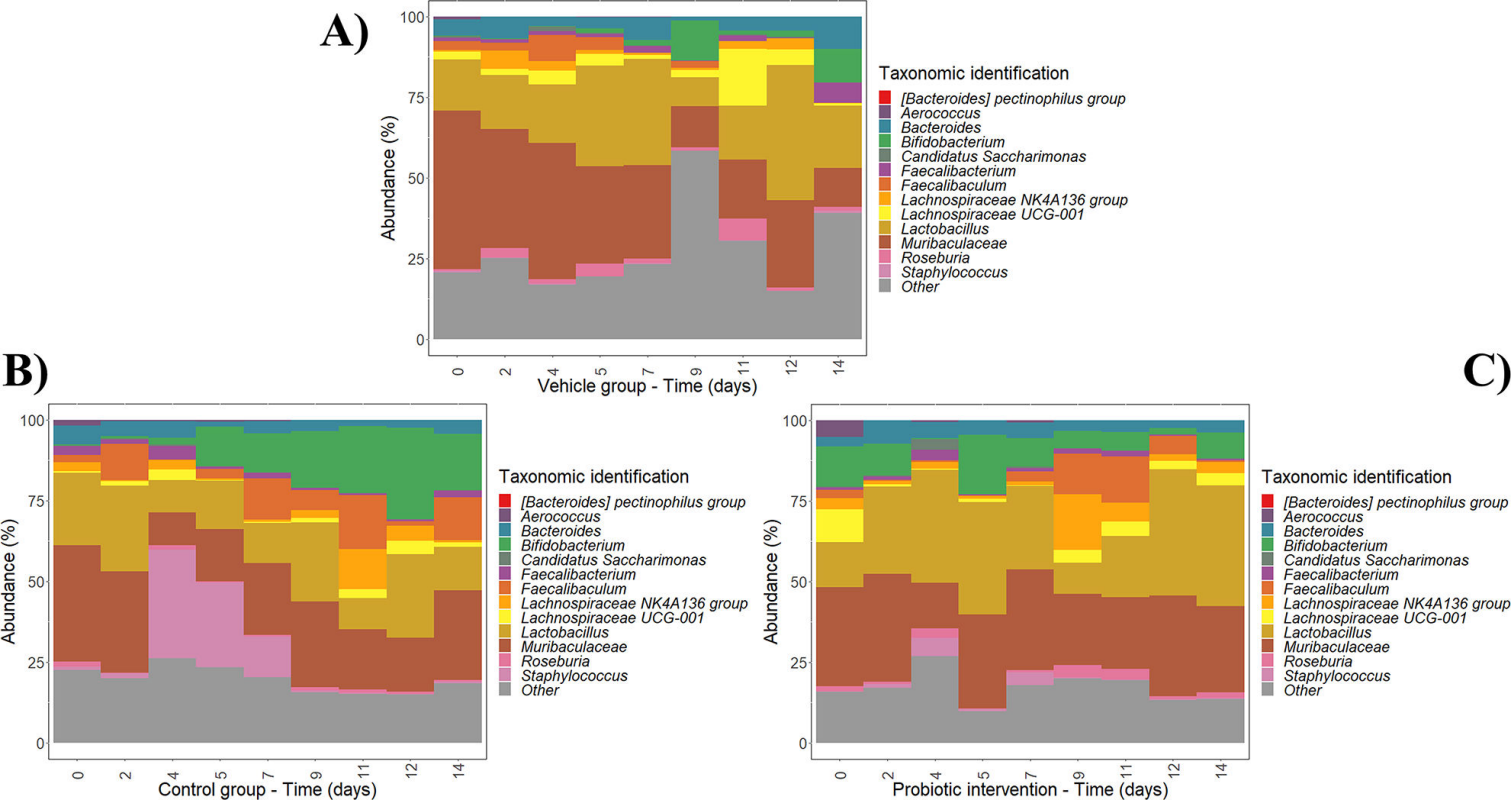
Changes in fecal microbiota genera from different groups: vehicle group (**A**); control strain, LMG10502_T_ (**B**); and GABA-producing strain, IPLA60004 (**C**) at different intervention times (0, 2, 4, 5, 7, 9, 11, 12, and 14 days). Taxa labeled as “Other” include the sum of taxa that did not show relevant changes at genus level. Data are expressed as abundance percentages (%).

In this regard, changes in fecal microbiota samples within each experimental group (vehicle group, control strain-non-GABA producer, and probiotic strain-GABA producer) were first determined at different times along the intervention period (0, 2, 4, 5, 7, 9, 11, 12, and 14 days). The abundances of microbial clades at phylum, family, and genus level showing differences along the intervention within each experimental group are illustrated in Fig. 3; Fig. S1 and S2.

With regard to the vehicle group (Fig. 3A; Fig. S1A and S2A), some taxa showed the highest abundance at 0 days, including Bacteroidota phylum, Muribaculaceae and Aerococcaceae families, and *Aerococcus* genus, while their representation was reduced along the experimental period. In contrast, the clades that were increased during the intervention include *Lachnospiraceae* NK4A136 group (maximum abundance at 2 days), *Faecalibaculum* (maximum abundance at 2 days), *Bifidobacterium* (maximum abundance at 9 days), *Lachnospiraceae* UCG-001 and *Roseburia* (maximum abundance at 11 days), *Lactobacillus* (maximum abundance at 12 days), and *Bacteroides* and *Faecalibacterium* (maximum abundance at 14 days).

With regard to the control strain group, which received daily the non-GABA-producer strain LMG10502^T^ (Fig. 3B; Fig. S1B and S2B), Bacteroidota phylum, *Muribaculaceae,* and *Aerococcus* showed the highest abundance at 0 days similar to the vehicle group, as well as *Bacteroides* and *Roseburia*. Clades increased during the intervention include *Lactobacillus* (maximum abundance at 2 days), *Faecalibacterium* (maximum abundance at 4 days), *Faecalibaculum* and *Lachnospiraceae* NK4A136 group (maximum abundance at 11 days), and *Bifidobacterium* and *Lachnospiraceae* UCG-001 (maximum abundance at 12 days).

Finally, the probiotic strain group (Fig. 3C; Fig. S1C and S2C) showed the highest abundance of *Aerococcus* at 0 days similar to the other groups. Clades that were increased during the administration of the probiotic strain include *Faecalibacterium* (maximum abundance at 2 days), *Bifidobacterium* (maximum abundance at 5 days), *Lachnospiraceae* NK4A136 group and *Roseburia* (maximum abundance at 9 days), *Faecalibaculum* (maximum abundance at 11 days), and *Lactobacillus* (maximum abundance at 12 days).

As it can be seen, similar clades were reduced and increased during the intervention in all groups. However, the group receiving the GABA-producer strain IPLA60004 showed higher abundances of *Lactobacillus* and *Roseburia* at late intervention times as compared to vehicle and control strain groups. In addition, the administration of the probiotic strain led to an increase in novel genera like *Lachnospiraceae* NK4A136 at late intervention times.

Remarkably, at late intervention periods, the representation of *Bifidobacterium* was higher in the groups receiving IPLA 60004 or LMG10502^T^ strains as compared to the group receiving only the vehicle.

### Microbiota profiling of colonic contents at the end of the intervention period

A complementary metataxonomic analysis of colonic contents taken at the end of the experimental period, following 14 days of intervention, was carried out. For this purpose, microbial diversity estimators were first computed. Alpha diversity coefficients measuring variability of genera within samples were calculated including Chao1 index. The mean Chao1 index of colonic content samples from vehicle (46.6 ± 9.7), control (46.9 ± 4.9), and probiotic (45.8 ± 6.6) groups was calculated at genus level, showing no major differences (Fig. S3A). Additional alpha diversity estimators including Shannon, Simpson, and Inverse Simpson indices (Fig. S3A) were calculated and compared, showing similar patterns in all the groups (Fig. S3B). These results also highlight the interindividual variability within groups. In general, the vehicle group showed higher alpha-diversity values than other groups, although no significant differences (*P* > 0.05) in alpha diversity estimators among groups were observed.

**Fig 4:**
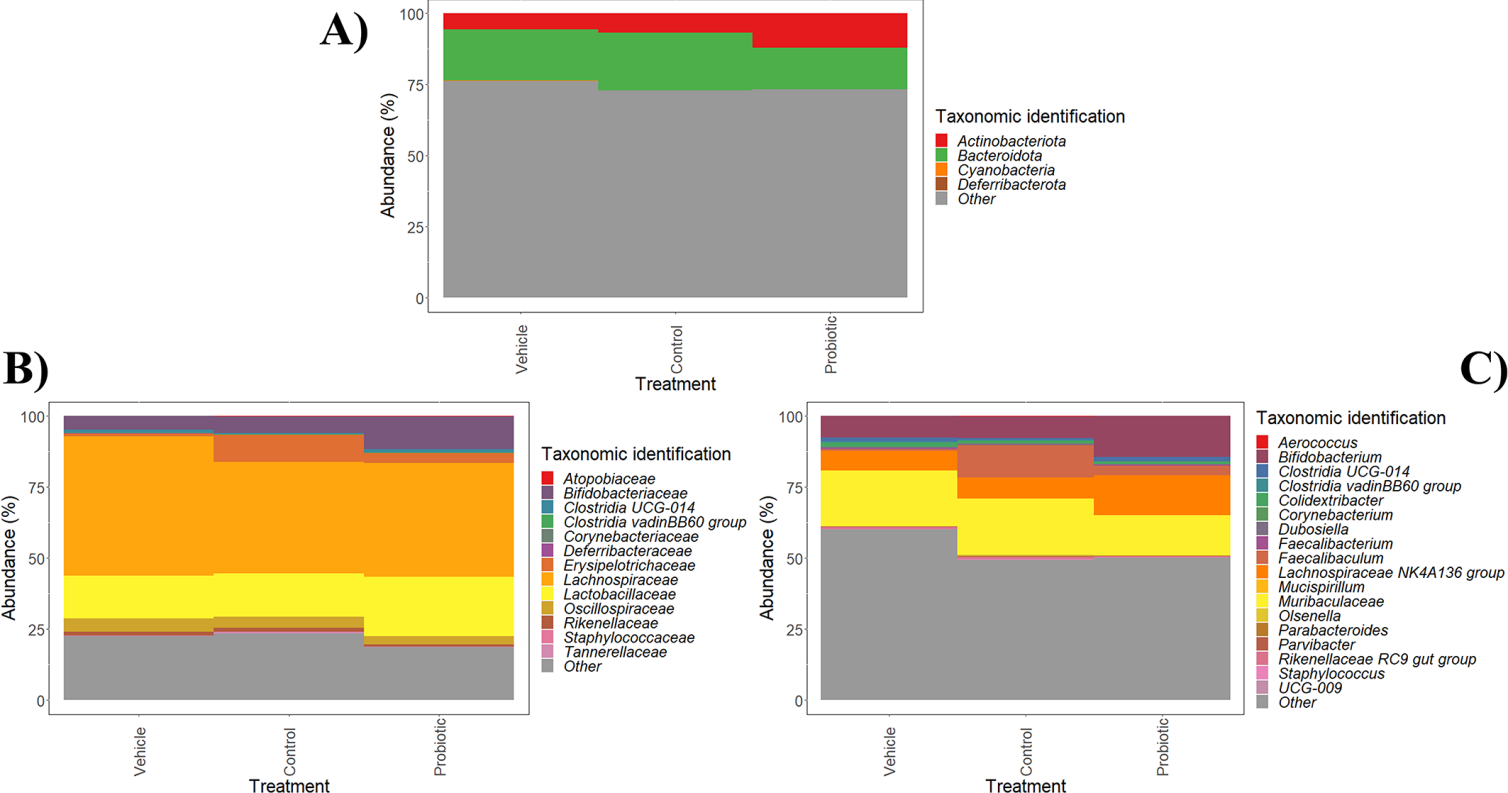
Changes in colonic content microbiota from different experimental groups (vehicle group, control strain, and probiotic strain groups) at phylum (**A**), family (**B**), and genus (**C**) level. Taxa labeled as “Other” include the sum of taxa that did not show any significant changes among the three groups. Data are expressed as abundance percentages (%).

Then, beta diversity analysis of samples, reflecting differences in microbial diversity between samples, was computed through Bray-Curtis dissimilarity metrics calculation (Fig. S4). Probiotic strain group showed higher diversity values at the phylum level compared to the other groups (Fig. S4A), while control and probiotic strain groups showed higher diversity values than the vehicle group at family and genus levels (Fig. S4B and C). However, the only significant (*P* < 0.05) differences were observed at family level (Fig. S4B). Bray-Curtis dissimilarity metric was used to compute PCoA of samples at phylum (Fig. S5A), family (Fig. S5B), and genus level (Fig. S5C), where samples from different groups could not be discriminated, highlighting the role of intragroup variability and indicating that possible taxa showing differences associated with the impact of IPLA60004 administration may account for discrete taxa only. However, significant changes in fecal microbiota from different groups at different intervention times were determined (Table 1; Fig. 3; Fig. S1 and S2). Furthermore, Bray-Curtis beta diversity distances were used to generate a cluster of colonic content samples (Fig. S6), revealing that most samples from the control and probiotic strain groups clustered in different branches at different taxonomic levels: phylum, family, and genus. These results highlight different microbiota profiles for control and probiotic strain groups. Multiple branches generated for each intervention group are due to the high intragroup variability of samples. Foremost, significant differences in colonic content microbiota from different intervention groups were reported (Fig. 4). Noteworthy, samples from control and probiotic groups showed higher dispersion in the PCoA, in agreement with the beta-diversity calculations (Fig. S4). In relation to the specific taxa that display different behavior in different groups, some of the changes in the microbiota upon administration of IPLA60004 exhibited higher magnitude as compared to the other groups, as is the case of *Lachnospiraceae* NKA136 group and *Bifidobacterium* whose increments were higher in the IPLA60004 group; or *Oscillospiraceae, Clostridia* vadinBB60 group, or *Rikenellaceae* that achieved the lowest abundance in the IPLA60004 group. In addition, significant differences in colonic content microbiota from different intervention groups were reported (Fig. 4), *Lachnospiraceae* and *Bifidobacterium* being the groups more strongly promoted in the IPLA60004 group as compared to the other two groups.

**TABLE 1:** Mean relative abundance of taxa determined by means of 16S rRNA metataxonomic profiling of colonic contents showing statistically significant differences among treatment groups (receiving vehicle, control strain LMG10502^T^, and GABA-producing strain IPLA60004) at phylum, family, and genus level

Taxa	Higher in	Vehicle		Control		Probiotic	
		Mean	SD	Mean	SD	Mean	SD
Phylum level							
Deferribacterota	Vehicle	0.06	0.06	0.04	0.05	0.03	0.04
Bacteroidota	LMG10502^T^	18.08	4.82	20.32	6.42	14.52	4.99
Cyanobacteria	LMG10502^T^	0.01	0.02	0.03	0.09	0.00	0.00
Actinobacteriota	IPLA60004	5.57	3.93	6.70	7.07	12.13	12.16
Family level							
Lachnospiraceae	IPLA60004	48.99	5.18	39.34	7.75	40.14	9.60
Oscillospiraceae	IPLA60004	4.72	0.91	3.86	0.95	2.94	1.30
Deferribacteraceae	IPLA60004	0.07	0.06	0.05	0.05	0.03	0.04
Clostridia vadinBB60 group	IPLA60004	0.24	0.15	0.16	0.23	0.10	0.12
Staphylococcaceae	LMG10502^T^	0.02	0.03	0.17	0.07	0.09	0.16
Erysipelotrichaceae	LMG10502^T^	0.97	1.44	9.42	13.42	3.42	6.73
Rikenellaceae	LMG10502^T^	1.35	0.53	1.35	0.84	0.75	0.56
Atopobiaceae	LMG10502^T^	0.01	0.01	0.17	0.22	0.17	0.26
Tannerellaceae	LMG10502^T^	0.19	0.16	0.36	0.22	0.08	0.06
Corynebacteriaceae	LMG10502^T^	0.00	0.00	0.06	0.07	0.04	0.10
Lactobacillaceae	IPLA60004	15.09	7.89	15.31	8.66	20.88	9.43
Bifidobacteriaceae	IPLA60004	4.87	4.00	5.80	7.20	11.56	13.04
Clostridia UCG-014	IPLA60004	0.95	0.58	0.41	0.20	1.15	1.06
Genus level							
*Faecalibacterium*	IPLA60004	0.46	0.30	0.14	0.14	0.29	0.43
*Colidextribacter*	IPLA60004	1.57	0.45	0.89	0.30	0.89	0.59
*Mucispirillum*	IPLA60004	0.10	0.10	0.06	0.07	0.04	0.05
*Parvibacter*	IPLA60004	0.08	0.10	0.01	0.02	0.06	0.07
*Clostridia vadinBB60* group	IPLA60004	0.37	0.25	0.26	0.38	0.12	0.15
*Butyricicoccaceae* UCG-009	IPLA60004	0.09	0.08	0.08	0.06	0.04	0.04
*Staphylococcus*	LMG10502^T^	0.03	0.04	0.25	0.11	0.11	0.19
*Faecalibaculum*	LMG10502^T^	0.61	1.59	11.21	16.48	3.24	7.58
*Muribaculaceae*	LMG10502^T^	19.43	5.70	19.81	5.07	14.18	5.02
*Rikenellaceae* RC9 gut group	LMG10502^T^	0.69	0.40	0.80	0.79	0.31	0.34
*Dubosiella*	LMG10502^T^	0.28	0.75	0.63	1.08	0.24	0.32
*Olsenella*	LMG10502^T^	0.01	0.02	0.23	0.30	0.20	0.30
*Aerococcus*	LMG10502^T^	0.00	0.00	0.02	0.03	0.00	0.01
*Parabacteroides*	LMG10502^T^	0.30	0.26	0.49	0.28	0.10	0.07
*Corynebacterium*	LMG10502^T^	0.00	0.00	0.09	0.10	0.06	0.16
*Bifidobacterium*	IPLA60004	7.49	6.37	7.75	9.25	14.36	15.50
*Lachnospiraceae* NK4A136 group	IPLA60004	6.90	5.88	7.37	5.73	14.08	8.91
*Clostridia* UCG-014	IPLA60004	1.46	0.92	0.59	0.30	1.51	1.48

To gain a better understanding of the impact of administering control (non-GABA producer) or probiotic strains (GABA producer) on specific members of the mouse microbiota, differences among colonic content samples (time 14 days) from different groups (vehicle, control strain, and probiotic strain) were determined (Table 1; Fig. 4). Vehicle group showed higher abundances of Lachnospiraceae and Oscillospiraceae families and *Faecalibacterium* and *Colidextribacter* genera compared to control and probiotic strain groups (Fig. 4). In addition, the abundance of Bacteroidota phylum, Tannerellaceae family, and an unidentified *Muribaculaceae* genus was higher in vehicle and control strain groups than in probiotic strain group. On the other hand, control strain group showed higher abundances of Staphylococcaceae and Erysipelotrichaceae families and *Staphylococcus*, *Faecalibaculum,* and *Parabacteroides* genera. In contrast, the administration of probiotic strain led to an increase in *Lactobacillaceae*, *Bifidobacterium,* and novel genera *Lachnospiraceae* NK4A136 group and *Clostridia* UCG-014 (Fig. 4).

### Correlation of colonic microbiota profiles, SCFA and GABA metabolites

**Fig 5:**
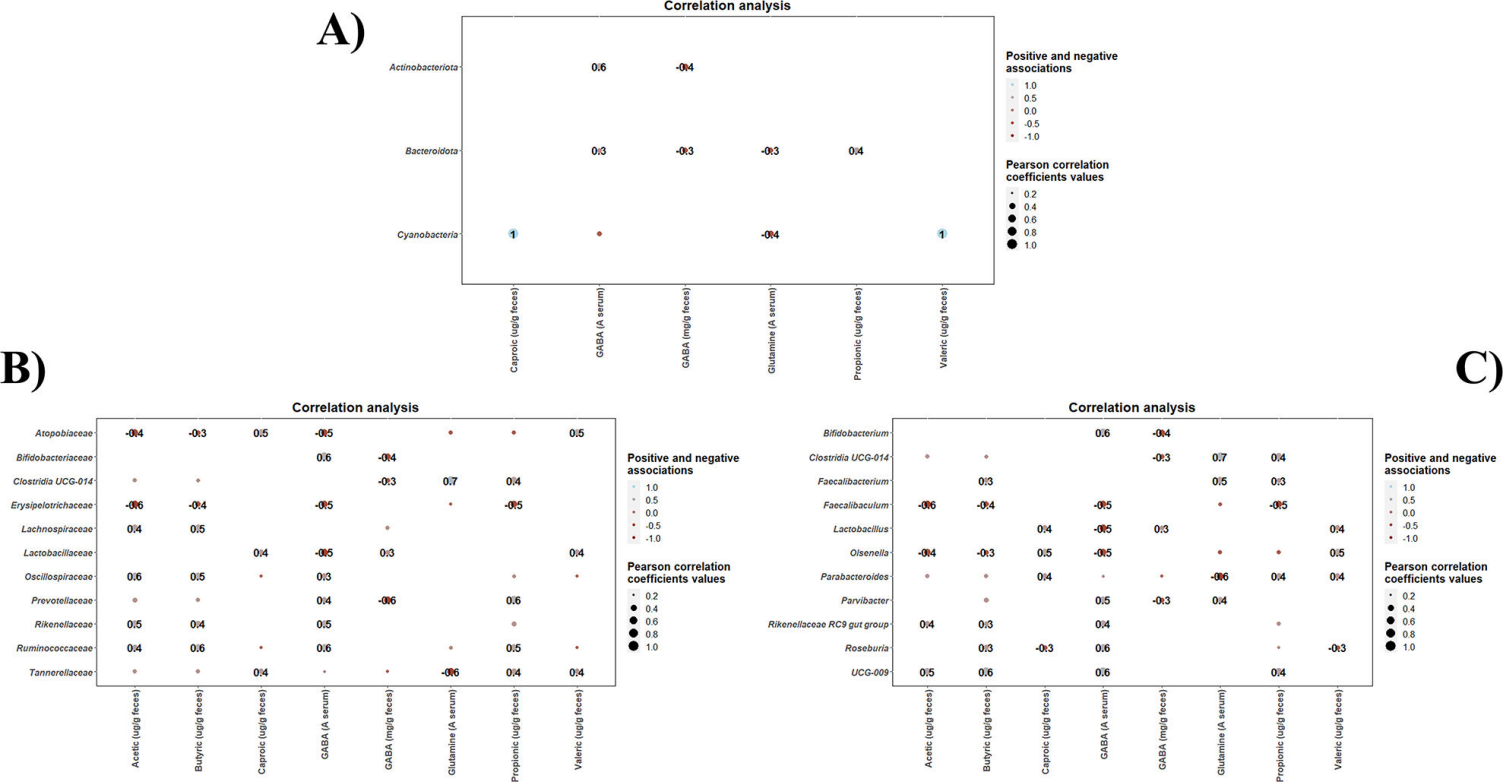
Correlation heatmaps showing the associations between taxonomic clades promoted by each intervention (vehicle group, control strain, and probiotic strain groups) and biochemical parameters (SCFAs, GABA, and glutamine levels). These correlations were determined at phylum (**A**), family (**B**), and genus (**C**) level. Blue and red dots indicate positive and negative correlations expressed as Pearson correlation coefficients. Color intensity and dot size are in proportion to magnitude. Fecal GABA is expressed as mg/g feces, while serum GABA was expressed as integrated signal area, A.

Statistical correlations between taxonomic clades promoted by each intervention and biochemical parameters in colonic contents (SCFAs, GABA, and glutamine levels) were detected (Fig. 5). As it can be seen, positive associations between Actinobacteriota phylum and serum GABA levels, and between Cyanobacteria and caproic and valeric acid levels, have been determined (Fig. 5A). At family level, several families (Bifidobactertiaceae, Oscillospiraceae, Prevotellaceae, Rikenellaceae, and Ruminococcaceae) showed positive associations with several SCFAs and/or serum GABA levels (Fig. 5B). In contrast, Tannerellaceae was negatively correlated with serum glutamine levels. Similar positive associations were found between different genera (*Parabacteroides*, *Parvibacter*, *Rikenellaceae* RC9 group, *Roseburia,* and novel *Butyricicoccaceae* genus UCG-009) and SCFAs and serum GABA levels (Fig. 5C). *Clostridum, Faecalibacterium,* and *Parvibacter* also correlated positively with serum glutamine levels (Fig. 5C). No statistically significant correlations were found among the taxa exhibiting modulation during the interventions and the colonic content of glutamate.

With regard to statistical correlations specifically determined within each intervention group, positive high correlations between *Bifidobacterium* and serum GABA levels determined in both control and probiotic strain groups but not in vehicle group (Fig. S7B and C). Similarly, *Butyricicoccaceae* UCG-009 and *Roseburia* were positively correlated with serum GABA levels in control strain group and probiotic strain group, respectively. In addition, *Bifidobacterium, Butyricicoccaceae* UCG-009, *Clostridia* UCG-014, and *Lactobacillus* showed positive correlations with several SCFAs in control strain group and probiotic strain group (Fig. S7B and C).

## DISCUSSION

In recent years, GABA production has emerged as a key trait mediating gut microbiome-brain signaling, and thus, increasing investigations have explored the contribution of GABA-producing probiotic strains to the amelioration of various conditions associated with metabolic and neuroendocrine disorders related to GABA disbalances (12, 28, 43, 44), or their capacity to transiently colonize and/or produce GABA at intestinal level (19). Since some of these disorders are frequently associated with gut microbiome disbalance, understanding the contribution of GABA-producing probiotics to gut microbiome modulation is a relevant aspect to understand their contribution to gut-brain signaling, yet only a few investigations analyzed the possible gut microbiota modulation effects of the administration of GABA-producing strains (44).

In this work, the administration of *B. adolescentis* strains, including the GABA producer IPLA 60004 previously characterized in our research group (29, 30) to healthy mice, did result in a significant alteration in the representation of several microbial taxa at intestinal level. Administration of control strain (LMG10502^T^) resulted in an increased representation in the colonic contents of *Faecalibaculum,* and administration of GABA-producing strain (IPLA60004) resulted in an increased representation of *Bifidobacterium*, *Lactobacilli*, and *Clostridia* UCG-014, as compared to the groups receiving only the vehicle. Remarkably, maximum increments of *Bifidobacterium* were achieved earlier during the intervention period, in the group receiving IPLA60004 as compared to the group receiving the control strain, LMG10502^T^, suggesting a differential colonization ability of both strains. These microbiome profiling results of the colonic contents at the end of the intervention period agree with those obtained in the longitudinal analysis of fecal samples. It is, however, worth noting that the number of taxa exhibiting significant differences among intervention groups was higher in fecal samples than in colonic contents. Prior works have demonstrated the existence of significant differences in the microbiota profiles of fecal and colonic contents in mouse models with colonic microbiota generally correlating better with disease biomarkers (45). Indeed, fecal samples only reflect the microbiota at distal colon sites, whereas in mice, microbial fermentation is known to occur predominantly at the cecum, close to proximal colon, and hence, its microbiota is not properly inferred from fecal microbiota analyses (46). Remarkably, in our work, some of the microbiota changes detected among intervention groups were detected in both colonic and fecal microbiota profiles.

The increased representation of *Bifidobacterium* in the control and probiotic groups was also consistent with the level of recovery of viable bifidobacterial cells in fecal samples, which reached levels up to 10^6^ CFU/mL along the intervention period for the groups receiving bifidobacterial strains but remained below the detection level (<10^2^ CFU/mL) for the vehicle group. In this regard, the level of fecal shedding of bifidobacteria during the intervention period agrees with those determined for mouse intervention assays with other bifidobacterial strains (19, 47, 48).

It is also worth noting that, according to fecal microbiota profiles, maximum relative abundances of bifidobacterial strains occurred earlier in the group receiving IPLA60004 (day 5), as compared to the control (day 12) and vehicle groups (day 9), which associates with higher relative abundances of *Bifidobacterium* in colonic contents in the group receiving the IPLA60004 strain. These observations contrast with results from fecal microbiota profiles, where relative abundances of the genus *Bifidobacterium* seem to reach higher values in the group receiving the control strain, LMG10502^T^, than in the IPLA60004 group, likely as a result of different overall fecal microbiota composition in both groups. Since 16S rRNA regions sequenced do not permit to discriminate among *Bifidobacterium* species, we cannot rule out the possibility that other bifidobacterial species naturally encountered in the animals might be contributing to the high levels of *Bifidobacterium* detected in the control group or even to those detected in the vehicle group. However, our culture-dependent results did not reveal differences in the amount of viable absolute amount of bifidobacterial cells that can be recovered from both control and probiotic groups and confirm the absence of recoverable bifidobacterial cells in the vehicle group, which were beyond the detection limit (<10^2^ CFU/mL) (30).

These observations confirm the existence of a strain-dependent impact on the mouse intestinal microbiome of *B. adolescentis* administration, in agreement with prior observations with other bifidobacterial strains (49, 50). Undoubtedly, the best control strain to study the specific contribution of the GABA-producing capacity of strain IPLA60004 to the gut microbiome modulation would have been a genetically modified strain, lacking the genes responsible for GABA production but genetically identical in rest of the attributes. Unfortunately, genetic modification tools are not readily available for most bifidobacterial strains, and, despite our efforts to achieve such comparator strain, we did not succeed. In view of this limitation, we employed as comparator strain the reference *B. adolescentis* LMG10502^T^ strain, which has been demonstrated not to harbor the *gad* genes (19). Notably, despite the genetic diversity encountered among *B. adolescentis* strains (51), prior comparative genomic and phenotypic analyses demonstrated no major genetic differences between LMG10502^T^ and IPLA60004 apart from the presence of the *gad* genes (29).

No differences in GABA metabolites were found in colonic contents of the animals receiving the GABA-producer strain, as compared to the groups receiving vehicle and control strain. This agrees with results from a prior work with other GABA-producing *B. adolescentis* strains, in which increases in GABA content in feces were very discrete, although expression of gadB/gadC genes was confirmed (19). These results with GABA-producing strains contrast with results obtained following the administration of other GABA-producing microorganisms such as *Lactobacillus brevis* strains (44) or recombinant *Bifidobacterium breve* strains heterologously expressing *gad* genes (12), although the level of GABA produced by such strains even *in vitro* is significantly higher than the one achieved by the native IPLA60004 strain. Despite this, *B. adolescentis* offers the advantage of holding the QPS status by EFSA and of being one of the bifidobacterial species most commonly found in the healthy human gut microbiota. Furthermore, although no differences in GABA concentrations in the colonic contents were detected, in the groups receiving bifidobacterial strains, we have identified a statistically significant association between the representation of bifidobacteria in colonic contents of the animals and the GABA concentrations determined at serum level for the same animals.

Although administration of the GABA-producing IPLA60004 strain does not directly correlate with increased fecal excretion of GABA metabolites of the animals, we cannot rule out an effect of the strain in the levels of the neurotransmitter at serum level. Indeed, GABA produced microbially at intestinal level could undergo several fate processes apart from direct fecal excretion. On the one hand, it could serve to sustain cross-feeding mechanisms among gut microbial strains (52); it could serve as a precursor for microbial degradation and production of other metabolites such as propionate (53); it could activate GABAergic receptors at intestinal level resulting in local and even systemic effects (14), or it could be transported and transferred to systemic circulation (54). In this sense, the serum GABA levels correlated positively with the representation of bifidobacteria in colon contents but also with the representation of other taxa such as *Ruminococcus*, which encompass species with the capacity to produce glutamate, a GABA precursor (55). Furthermore, administration of the GABA-producing IPLA60004 strain resulted in a modulation of other accompanying members of the microbiota, some of which could contribute to GABA metabolism and production. Indeed, the group receiving the IPLA60004 strain exhibits significant changes in the representation of several other gut microbiota species, including Lachnospiraceae, lactobacilli, and some clostridia species and altered production of SCFA, reflected by a reduced content of total SCFAs and, particularly, to lower contents of butyric and acetic acid. Indeed, *B. adolescentis* has been reported to produce only significant amounts acetic acid, yet this can serve to sustain cross-feeding relationship with other accompanying members of the microbiota (56). Remarkably, several gut commensal species apart from bifidobacteria correlated positively with serum GABA levels. Among these is very notable the presence of several commensal and beneficial taxa, including *Roseburia*, a well-known butyrate producer that includes species proposed as potential psychobiotics (57) and with the capacity to produce serotonin (58).

In conclusion, administration of the GABA-producing *B. adolescentis* IPLA60004 strain resulted in significant modulation of a range of taxa in the intestinal microbiome of mice, different from the modulation exerted by non-GABA producers. Although the administration of IPL60004 did not result in increased content of GABA-related metabolites at intestinal level, intestinal content of bifidobacterial cells positively correlated with the serum content of GABA. To our knowledge, this is the first investigation into the potential gut microbiome modulatory effects of the administration of a GABA-producing *B. adolescentis* strain. Further investigation on the functional and metabolic routes modulated in both the microbiota and the host will undoubtedly contribute to delineate the specific mechanisms by which IPLA60004 administration contributes to reduce serum glutamate levels and to ascertain whether this effect could have a health benefit in human patients of diseases associated with high-glutamate serum concentrations.

## Data Availability

The reads generated in this study have been deposited in the NCBI Sequence Read Archive (SRA) under Bioproject accession number PRJNA956357 and sample codes SAMN34211560 and SAMN34211484.

## SUPPLEMENTAL MATERIAL

Supplemental figures spectrum.02580-23-s0001.docx Figures S1 to S7.

ASM does not own the copyrights to Supplemental Material that may be linked to, or accessed through, an article. The authors have granted ASM a non-exclusive, world-wide license to publish the Supplemental Material files. Please contact the corresponding author directly for reuse.
