# Supplementary material for: Mouse intestinal microbiome modulation by oral administration of a GABA-producing Bifidobacterium adolescentis strain
Source: Microbiol Spectr. 2023 Nov 22;12(1):e02580-23. doi: 10.1128/spectrum.02580-23 (PMC10783132; doi:10.1128/spectrum.02580-23)

**Mice intestinal microbiome modulation by oral administration of a GABA-producing *Bifidobacterium adolescentis* strain*.***

Héctor Tamés ^1,2&^, Carlos Sabater^1,2&^, Félix Royo^3,4,5^, Abelardo Margolles^1,2^, Juan Manuel Falcón^3,4,5^, Patricia Ruas-Madiedo^1,2#^, Lorena Ruiz^1,2#^

^1^ Department of Microbiology and Biochemistry of Dairy Products, Instituto de Productos Lácteos de Asturias-Consejo Superior de Investigaciones Científicas (IPLA-CSIC), Paseo Río Linares s/n, 33300, Villaviciosa, Asturias, Spain.

^2^ Functionality and Ecology of Beneficial Microbes (MicroHealth) Group, Instituto de Investigación Sanitaria del Principado de Asturias (ISPA), Oviedo, Asturias, Spain

^3^ Exosomes Laboratory. Center for Cooperative Research in Biosciences (CIC bioGUNE), Basque Research and Technology Alliance (BRTA), 48160, Derio, Spain

^4^ Centro de Investigación Biomédica en Red de Enfermedades Hepáticas Y Digestivas (CIBERehd), 28029, Madrid, Spain.

^5^ IKERBASQUE, Basque Foundation for Science, Bilbao, 48011, Spain.

**Running title**: Microbiome modulation by GABA-producing *B. adolescentis*

**Keywords**: *B. adolescentis*; GABA; microbiome modulation;

^&^These authors have equally contributed to this work

***Corresponding author**: address: Instituto de Productos Lácteos de Asturias-Consejo Superior de Investigaciones Científicas (IPLA-CSIC), Paseo Río Linares s/n, 33300, Villaviciosa, Asturias, Spain; email: [lorena.ruiz@ipla.csic.es](mailto:lorena.ruiz@ipla.csic.es); phone: +34 985 89 21 31; ruas-madiedo@ipla.csic.es

**Supplementary Figure S1**. Changes in fecal microbiota phyla from different groups: vehicle group (**A**), control strain, LMG10502^T^ (**B**) and GABA-producing strain, IPLA60004 (**C**) groups at different intervention times (0, 2, 4, 5, 7, 9, 11, 12 and 14 days). These taxa constitute the core microbiota of samples. Taxa labelled as “Other” include the sum of taxa that did not show relevant changes at phylum level. Data are expressed as abundance percentages (%).

**Supplementary Figure S2**. Changes in fecal microbiota families from different groups: vehicle group (**A**), control strain, LMG10502^T^ (**B**) and GABA-producing strain, IPLA60004 (**C**) groups at different intervention times (0, 2, 4, 5, 7, 9, 11, 12 and 14 days). These taxa constitute the core microbiota of samples. Taxa labelled as “Other” include the sum of taxa that did not show relevant changes at family level. Data are expressed as abundance percentages (%).

**Supplementary Figure S3**. **A)** Comparison of different alpha-diversity indicators (Chao1, Shannon, Simpson and Inverse Simpson) of the relative abundance of taxa determined in the colonic content microbiota of samples from different intervention groups (vehicle group, control strain - LMG10502^T^-, and GABA-producing strain -IPLA60004-). These indicators measure the variability of microbial taxa within a sample. **B)** Relationship between different alpha-diversity estimators: Chao1, Shannon, Simpson and Inverse Simpson indices. These coefficients reflected similar patterns in the core microbiota.

**Figure S4**. Beta-diversity analysis of colonic content microbiota samples from different intervention groups (vehicle group, control strain - LMG10502^T^-, and GABA-producing strain -IPLA60004-) at phylum (**A**), family (**B**) and genus (**C**) level. These indicators measure the variability of microbial taxa between groups of samples. Bray-Curtis dissimilarity method was selected for the calculation. : a,b Statistically significant (p < 0.05) differences between intervention groups.

**Supplementary Figure S5**. Principal coordinates analysis (**PCoA**) of colonic content microbiota samples from different intervention groups (vehicle group, control strain - LMG10502^T^-, and GABA-producing strain -IPLA60004-) at phylum (**A**), family (**B**) and genus (**C**) level. Samples from the same intervention group could not be completely discriminated highlighting the role of individual variability. **PC:** principal coordinate. The percentage (%) of variance explained by each PC is indicated in the axis.

**Supplementary Figure S6.** Clustering of analysis of colonic content microbiota samples from different intervention groups (vehicle group, control strain and probiotic strains) at phylum (**A**), family (**B**) and genus (**C**) level. Cluster branches measure the variability of microbial taxa between groups of samples. Bray-Curtis dissimilarity method was selected for the calculation.


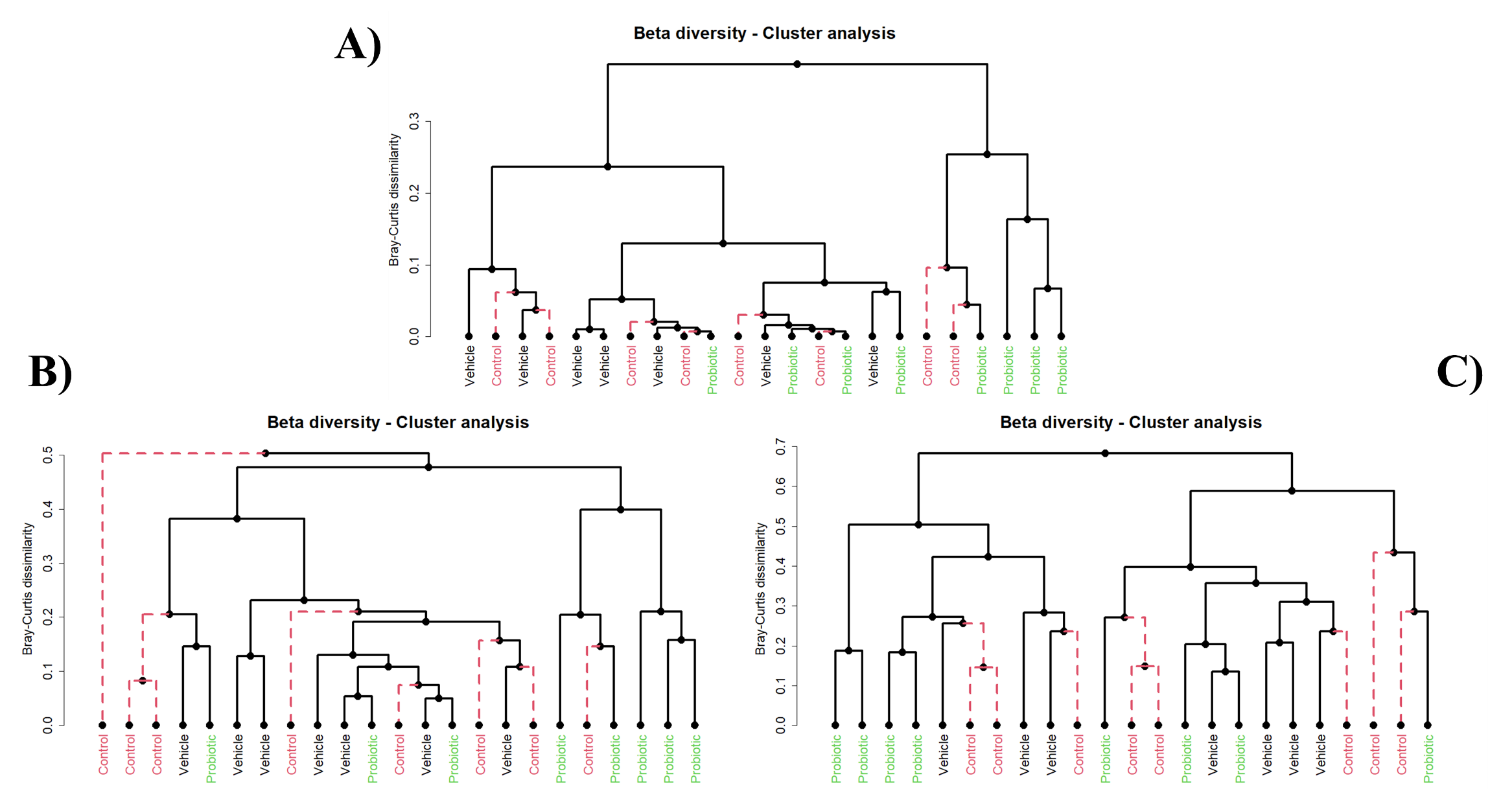


**Supplementary Figure S7.** Correlation heatmaps showing the associations between taxonomic clades promoted by each intervention (vehicle group, **A**; control strain, **B**; and probiotic strain, **C**; groups) and biochemical parameters (Short Chain Fatty Acids, SCFAs; gamma-amminobutyric acid, GABA; and glutamine levels). These correlations were determined at genus level. Blue and red dots indicate positive and negative correlations expressed as Pearson correlation coefficients. Color intensity and dot size are in proportion to magnitude. Fecal GABA is expressed as mg/g feces while serum GABA was expressed as integrated signal area.


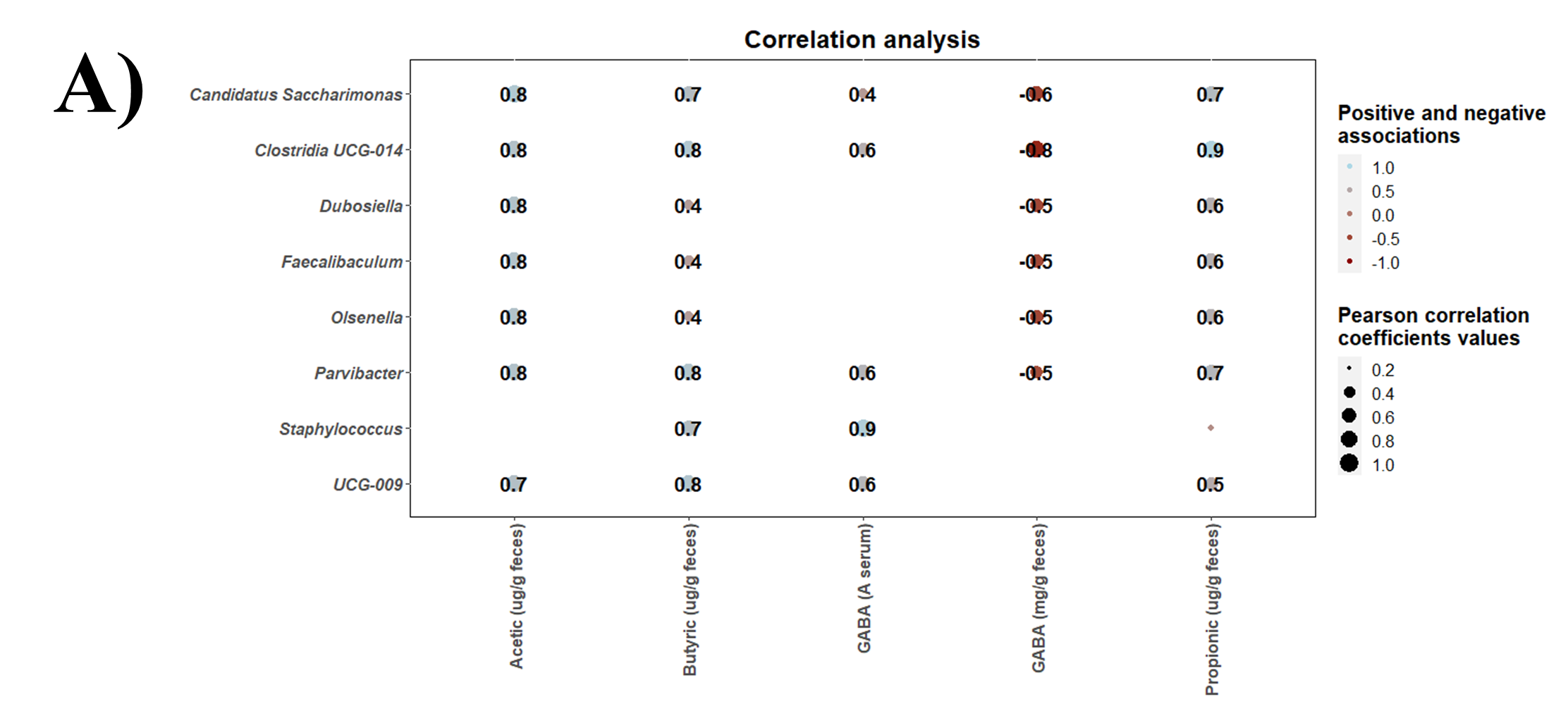


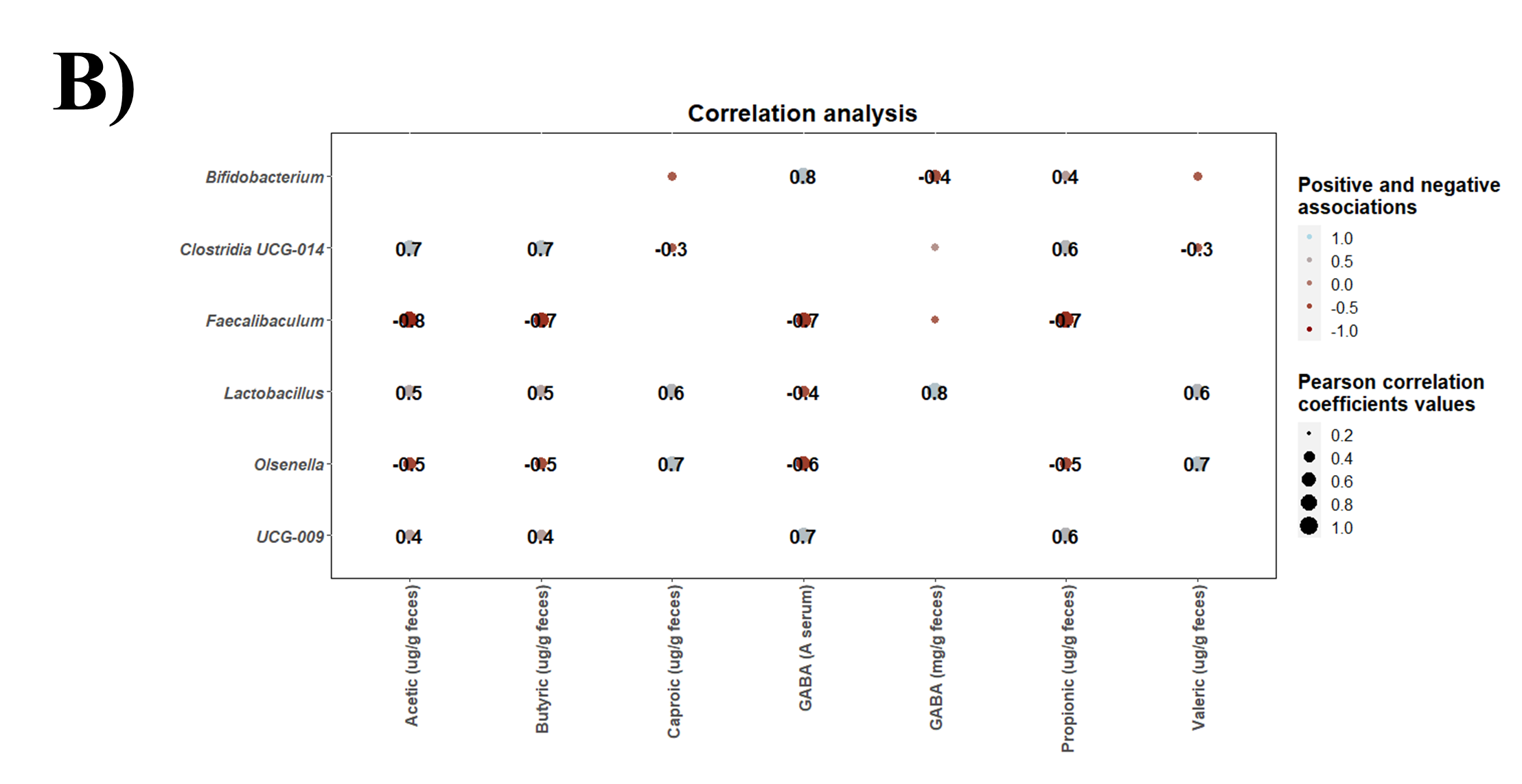


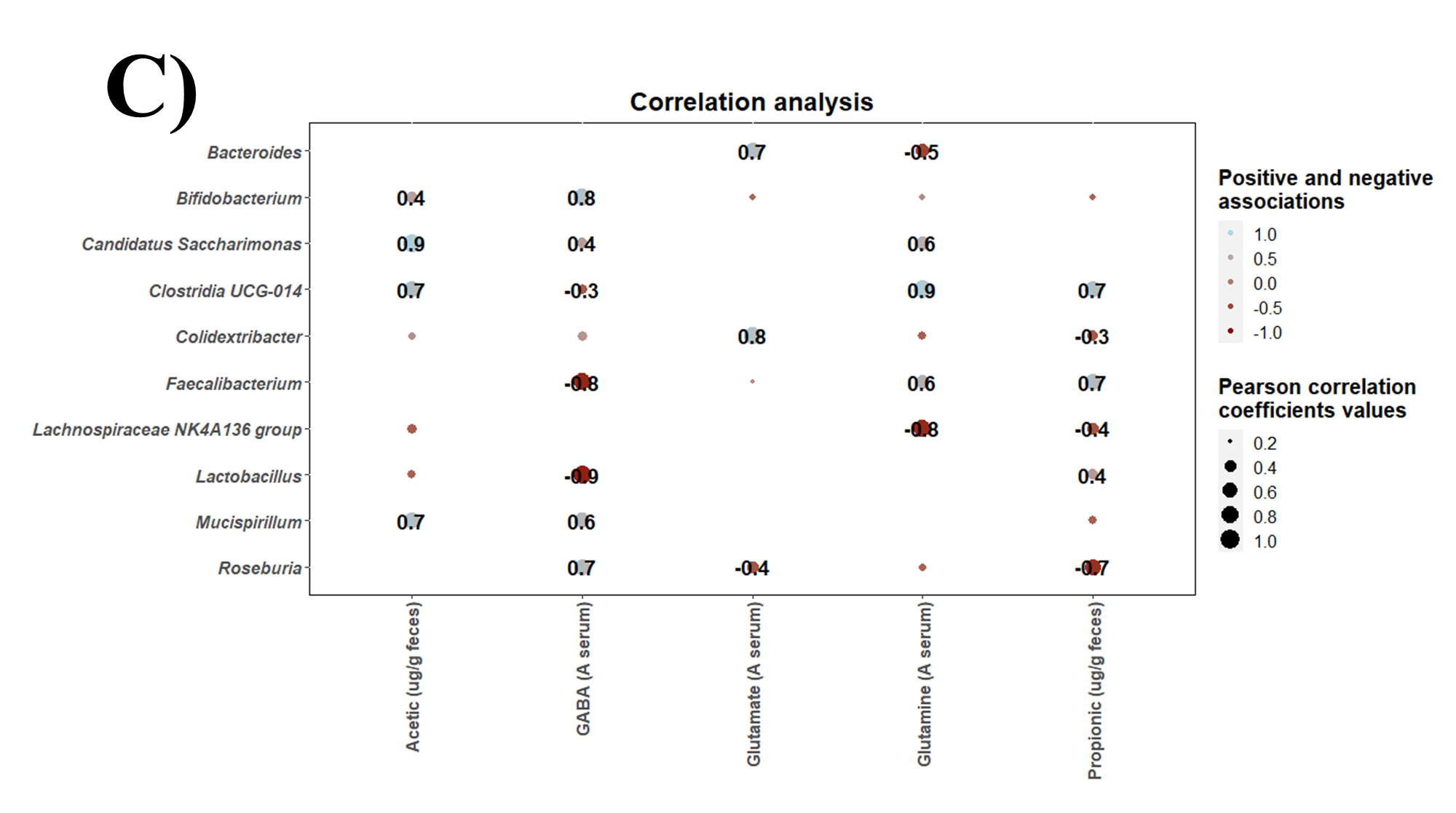

Supplement: Supplemental figures — Figures S1 to S7. [file spectrum.02580-23-s0001.docx]
